# 

**Published:** 1922-05

**Authors:** 


### ANNOUNCEMENTS

### Northern Ohio Dental Association

The sixty-fifth annual session of the Northern Ohio Dental Association will be held at Hotel Statler, Cleveland, Ohio, June 5, 6 and 7, with postgraduate course following. George B. Smith, Secretary, Fremont, Ohio.

### American Dental Society of Europe

The thirty-ninth meeting of the American Dental Society of Europe will be held in London, July 28-31, 1922. American members of the profession all over the world are cordially invited. William A. Spring, *President,* 8 W. 40th St., New York, N. Y.; F. W. Fleming, Secretary, 13 Queen Anne St., London, England.

### American Institute of Dental Teachers

The twenty-ninth annual meeting of the American Institute of Dental Teachers was held at Montreal, Quebec, January 23, 24 and 25. The following officers were elected to serve for the ensuing year: President, A. H. Hipple, Omaha, Neb.; Vice-President, A. E. Webster, Toronto, Ont.; Secretary-Treasurer, Abram Hoffman, Buffalo, N. Y. (re-elected); Members of the Executive Board, E. D. Coolidge, Chicago, Ill.; Herbert L. Wheeler, New York City; R. H. Volland, Iowa City, Iowa.

The next meeting will be held at Omaha, Nebraska, January 22, 23 and 24, 1925. Abram Hoffman, Secretary-Treasurer, 381 Lindwood Ave., Buffalo, N. Y.

### Dr. Henry D. Justi

Philadelphia, Pa., February 6.—Henry D. Justi, well known for his philanthropies and a pioneer in the dental supply business, died yesterday at his home, 3401 Baring

Street. He was eighty-eight years old. Death was due to a complication of diseases.

In 1857 Mr. Justi established a dental supply house at 516 Arch Street. As a result of the increase in business he subsequently moved to Thirteenth and Arch Streets. The present establishment at Thirty-second and Spring Garden Streets is conducted under the name of H. D. Justi & Son.

Mr. Justi was a member of the Lutheran Church and the Masonic fraternity. He is survived by a widow, Elizabeth C. Justi, a son, Henry M. Justi, and two daughters.

---

### Robert E. Hastings

Robert E. Hastings, senior member of The Morgan-Hastings Company, of Philadelphia, died January 31, 1922. Mr. Hastings was for fifty-seven years one of the best known gold manufacturers and was highly esteemed for his strict integrity and active interest in dental affairs.

---

### National Societies

National Dental Association, July 17-21, 1922, Ambassador Hotel, Los Angeles, Cal. Otto U. King, Secretary, 127 N. Dearborn St., Chicago, Ill.

National Society of Denture Prosthetists, July 3-15, Dental Department, University of Southern California, Los Angeles, Cal. E. B. Owen, Secretary, 335 Frisco Bldg., St. Louis, Mo.

National Association of Dental Examiners, July 17 and 18, Ambassador Hotel, Los Angeles, Cal.

National Association of Dental Faculties, July 13, 14, 15, Hotel Clark, Los Angeles, Cal.

National Dental Sorority, July 17, Ambassador Hotel, Los Angeles, Cal.

American Dental Library and Museum Association, July 17, Ambassador Hotel, Los Angeles, Cal.

American Society of Oral Surgeons and Exodontists, July 11-14, Hotel Alexandria, Los Angeles, Cal.

Association of Military Dental Surgeons, July 18, Los Angeles, Cal.

American Academy of Periodontology, July 10, 11, 1922, Drake Hotel, Chicago, Ill. J. Herbert Wood, Secretary, Cleveland, Ohio.

American Society of Orthodontists, April 24, 25, 26, 1922, Edgewater Beach Hotel, Chicago, Ill. Ralph Waldron, Secretary.

---

### New York Dental College Reunion

A reunion of the alumni of the New York College of Dentistry from 1867 to 1922 will be held on June 9, 1922. Clinics at the college during the day and a banquet at a prominent hotel in the evening are contemplated. Program to be announced later. Samuel H. Solomon, Secretary Publicity Committee, 1022 Trinity Ave., New York City.

---

### Co-operation of Public Health Workers in a Study of Public Health Education and Publicity

The Department of Surveys and Exhibits, Russell Sage Foundation, is making a study in social welfare education and publicity, with special reference to some phases of education in the health field.

The immediate studies deal with the qualifications, training and responsibilities of those who do the work; with the materials for education and publicity as supplied by national or state agencies or produced by local agencies; with the methods of all three groups in making use of these materials.

### CO-OPERATION INVITED

Any or all of the following will be of considerable value:

1. Outlines of "programs" or plans for publicity or education; detailed descriptions of publicity projects or of plans for using various forms of publicity and the committee organization for "putting over" an idea or item of information.

2. Samples of all forms of publicity; press stories; photographs and other material.

3. Names and dates of particular events, as "health days," "health weeks," or other campaigns, and any significant public meetings, or meetings for the discussion of publicity methods.

4. Names of persons who are doing particularly good work in health education or publicity through the use of graphic material, printed matter, making addresses, organizing meetings, making use of an automatic stereopticon or other equipment, object lessons in speaking, or otherwise.

5. Reports of observations on how people react to educational material of any kind or to any practical teaching methods.

6. Comments and criticisms on the effectiveness and suitability of educational or publicity material put out by national or state organizations in the health field.

7. Unsolved problems of those who are doing health educational or publicity work, as budgets, supervision of their work, opportunities for training, or whatever may affect efficiency or satisfaction in their work.

8. A place on all of your mailing lists for the following address: E. G. Routzahn, 130 East 22nd Street, New York City. E. G. Routzahn, Mary Swain Routzahn.

